# Aptamer-Based Colorimetric Probe for *trans*-Zeatin Detection Using Unmodified Gold Nanoparticle

**DOI:** 10.1155/2020/8853451

**Published:** 2020-10-26

**Authors:** Ping Sun, Xiwei Zhang, Xianxiang Wang

**Affiliations:** ^1^Information Materials and Device Applications Key Laboratory of Sichuan Provincial Universities, Chengdu University of Information Technology, Chengdu 610225, China; ^2^State Key Laboratory of Electronic Thin Films and Integrated Devices, University of Electronic Science and Technology of China, Chengdu 610054, China; ^3^College of Science, Sichuan Agricultural University, Chengdu 611130, China

## Abstract

*Trans*-Zeatin is the major active phytohormone in immature corn kernels. Herein, a highly sensitive, good selective and simple aptamer-based colorimetric method for the detection of *trans*-zeatin was constructed. The selected aptamer sequence binds with *trans*-zeatin and induces a duplex-to-aptamer structure switching. The gold nanoparticles (AuNPs) solution is stable with high-concentration salt, which is protected by red complementary DNA. In the absence of *trans*-zeatin, the color of AuNPs changed from red to blue because aptamer DNA and complementary DNA form double-stranded DNA. Thus, the ratio of absorbance intensities (A522/A650) of AuNPs is changed with the concentration of *trans*-zeatin. The color change could be observed by the naked eye. The linear range of this method covers a large variation of *trans*-zeatin concentration from 0.05 to 0.75 *μ*M. The detection limit is 0.037 *μ*M. Moreover, this method was applied successfully to detect *trans*-zeatin in real plant samples.

## 1. Introduction

*Trans*-Zeatin, 6-(4-hydroxy-3-methyl-2-transbutenylamino)purine is the major active phytohormone, which is discovered in immature corn kernels from the genus *Zea*. As one of the plant growth hormones, it plays a key role in controlling plant development and aging, including cell division, tissue differentiation, and plant growth [1, 2]. Especially, the levels of *trans*-zeatin have a large effect on plants. It can promote callus initiation, retard yellowing for vegetables and fruit after blossom, and cause auxiliary stems to grow and flower [3]. Hence, it is very important to detect the content of *trans*-zeatin in agricultural production. The current detection methods are still largely dependent on enzyme-linked immunosorbent assay (ELISA) [4–6] or high-performance liquid chromatography (HPLC) [7, 8]. The mechanism of ELISA is using zeatin as an antigen to react with specific labeled antibody. This method is highly sensitive, but it has high cost for expensive agents and time consuming for cockamamie operation steps. Meanwhile, overall recoveries of HPLC are not even 10% sometimes, which requires tedious preprocessing steps and expensive instrumentation [9]. It is necessary to find a simpler, more responsive, and easily operable method to measure the quantity of *trans*-zeatin in many agricultural fields.

Aptamers are selected in vitro through systematic evolution of ligands by exponential enrichment (SELEX). The artificial single-stranded DNA and RNA sequences that fold into secondary and tertiary structures make them bind to certain targets with extremely high specificity and affinity. They possess high recognition for small inorganic or organic substances, even proteins or cells [10–12]. Therefore, a different aptamer has been developed as a specific recognition probe for target. Many analytical methods were reported, such as fluorescence [13, 14], electrochemistry [15], chemiluminescence [16, 17], and colorimetric [18, 19]. Compared with the above methods, colorimetric is a convenient alternative method which can be carried out without any special expensive instrumentation and some costly reagents [20–23]. Gold nanoparticles (AuNPs) have been used widely as a colorimetric probe, due to the shift of surface plasmon resonance interfered by other ions or molecules. The color of dispersed AuNPs is red, and the aggregated is blue. Aptamers are single-stranded oligonucleotides (ssDNA or ssRNA). ssDNA with random coil structures could adsorb on the surface of AuNPs and protect AuNPs from salt-induced aggregation [24]. Because of distinctive adsorptive properties of the aptamers toward AuNPs, the binding of the target or conformational changes in the DNA result in assembly of the AuNPs to produce colorimetric signal [25].

In this study, a *trans*-zeatin's aptamer was used to protect AuNPs against salts. The AuNPs are applied as a colorimetric probe for *trans*-zeatin because the color of AuNPs will be changed from blue or purple to red with the concentration of *trans*-zeatin. Compared with the other methods, this colorimetric method allows qualitative detection of *trans*-zeatin in naked eye (without the aid of any instrument) in an uncomplicated and inexpensive manner with low concentration.

## 2. Experimental

### 2.1. Materials and Apparatus

*Trans*-Zeatin, adenosine, adenine, adenosine monophosphate, adenosine diphosphate, and adenosine triphosphate were bought from Sigma-Aldrich. HAuCl_4_·3H_2_O, sodium citrate, and other chemicals were purchased from Kelong Reagent Co., Chengdu, China. All reagents were of analytical grade and used without any further purification.

The *trans*-zeatin's aptamer with the sequence of 5′- CGG ATA TGG TTA GGC AGG CAT AAG AGG TTT ATC CG -3′ and its complementary DNA with the sequence of 5′- CGG ATA AAC CTC TTA TGC CTG CCT AAC CAT ATC CG -3′ was adopted from Qi et al.'s work [26]. They were synthesized by Sangon Biotech Co., Ltd. (Shanghai, China) and purified by the method of polyacrylamide gel electrophoresis. Di et al. have generated the aptamers against zeatin by SELEX technique and developed a simple and selective fluorescent sensor for zeatin by integrating graphene oxide (GO) [26]. The concentration of DNA was determined by measuring the UV-vis absorption at 260 nm with an extinction coefficient of 204,600 M^−1^·cm^−1^. All solutions were prepared with water purified by a Milli-Q Purification System (Millipore, USA). The UV-vis absorption spectra were performed with an Ultrospec 6300 pro-UV-vis spectrophotometer (Amersham Biosciences). Shimazu Prominence HPLC system (Shimadzu, Japan) was used to verify the accuracy of the method.

### 2.2. Preparation of AuNPs

Citrate-capped AuNPs were prepared according to the classical Frens' method [27]. In brief, 100 mL of 0.01% (w/w) HAuCl_4_ solution was heated to boiling for 2 min, and then 1 mL of 1% sodium citrate solution was added rapidly with vigorous stirring. The color of the mixed solution changed from pale yellow to deep red. The heating was stopped after reflux for another 15 min. Thereafter, the solution was cooled to room temperature while being stirred continuously. Finally, the cooled solution was diluted and stored in a refrigerator at 4°C for further use.

### 2.3. Analytical Procedures

1 *μ*L aptamer DNA solution (10 *μ*M) was mixed with different concentrations of *trans*-zeatin for *trans*-zeatin determination. The mixture was vortex-mixed thoroughly and incubated for 10 min. The *trans*-zeatin and aptamer would interact efficiently with each other. 1 *μ*L complementary DNA solution (10 *μ*M) was added in the mixed solution and incubated another 5 min. Afterwards, 1 *μ*L of the prepared hybridization solution was added in 100 *μ*L of AuNPs and incubated another 10 min. Finally, 4 *μ*L NaCl (0.2 M) was added in the mixed solution. The ratio of absorbance intensities (A522/A650) was measured to indicate the aggregation of the AuNPs using a spectrometer after 5 min. The control groups were measured in the same processing. All experiments were conducted at ambient temperature.

### 2.4. Application in Plant Samples

Leaves were obtained from 5-week-old seedlings grown in the greenhouse and were lyophilized for 48 h before analyses. *Trans*-zeatin was extracted from 10 g of leaves at −20°C overnight. The extraction process is as follows. 500 mL of 0.2 M acetic acid-methanol (80 : 20) and 100 mg/mL of butylated hydroxytoluene solution was added and mixed for 24 hours in the absence of light and −20°C. The extracts were filtered, centrifuged, and concentrated. Then, the pH of the extract was adjusted to 3 with 1 M sulfuric acid. The pigments were removed with ethyl acetate. Liquid exacts were purified with syringe filters and stored in the refrigerator for future use.

To compare the accuracy of this method, samples were tested by high-performance liquid chromatography (HPLC) with UV detector. The HPLC is equipped with a C18 250 mm × 4.6 mm (5 *μ*m) ODS-2 column. The mobile phase contains methanol-water (40 : 60) and acidified (5% acetic acid). The flow rate was set at 0.8 mL·min^−1^. Wavelength is 280 nm, and analysis time is 10 min. After establishing a standard curve with standard *trans*-zeatin, samples were measured, and the method was verified by standard addition method.

## 3. Results and Discussion

**Figure 1:**
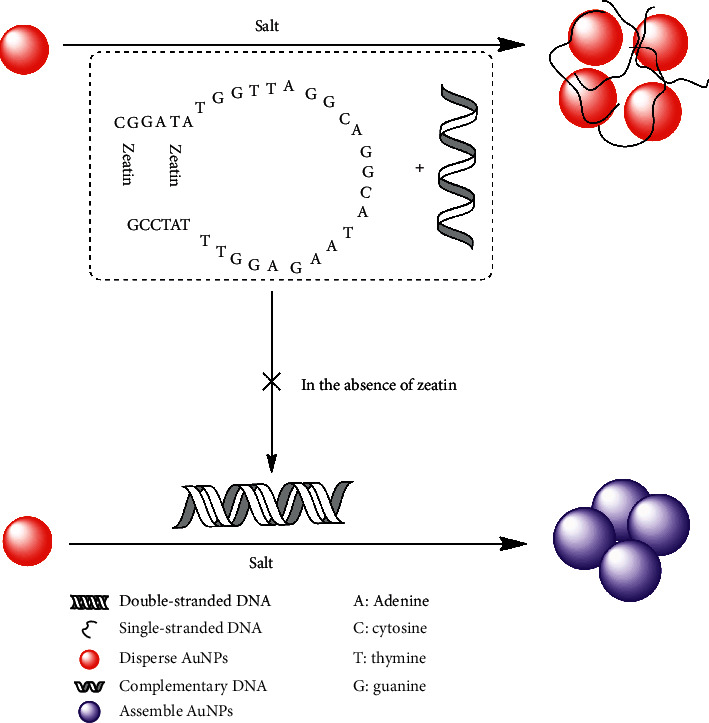
Schematic description of the AuNPs-aptamer-based strategy for *trans*-zeatin assay. In the presence of *trans*-zeatin, the binding of *trans*-zeatin to the aptamer sequence induces a duplex-to-aptamer structural switching. The AuNPs are stabilized by only complementary DNA, showing high resistance to salt-induced aggregation (solution staying in red). In the absence of *trans*-zeatin, aptamer DNA, and complementary DNA formed double-stranded DNA. AuNPs are not stabilized, and thus they are readily aggregated by salt (solution displaying blue colors).

**Figure 2:**
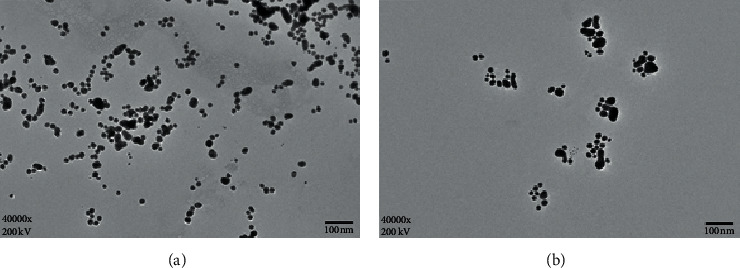
(a) The TEM images of AuNPs and (b) the AuNPs aggregate in the absence of a low concentration of *trans*-zeatin.

AuNPs's size-dependent surface resonance (SPR) absorption located at about 518 nm has been employed widely as a colorimetric sensor for many molecules [24, 28, 29]. The color of the gold solution reflects the degree of aggregation of AuNPs in suspension. The aggregation can be induced easily by salts. Due to the intermolecular forces and hydrophobic forces between the unmodified AuNPs and ssDNA, the strong interaction keeps them to combine tightly with each other to form an AuNPs-ssDNA conformation. The AuNPs are protected from aggregation in high-concentration salts.  Figure 1 shows the detection strategy for *trans*-zeatin with AuNPs. In the presence of *trans*-zeatin, the aptamer preferentially binds to *trans*-zeatin and assembles a rigid duplex, which leads to the added ssDNA strand (complementary DNA) release. The ssDNA spontaneously binds to unmodified AuNPs through interactions and stabilizes AuNPs effectively. So, the ssDNA-stabilized AuNPs show enhanced significant resistance to salt-induced aggregation (the color of solution is still red). Otherwise, in the absence of *trans*-zeatin, double-stranded DNA will form between aptamer and its complementary DNA. As a consequence, the AuNPs is not to be protected, and thus they are readily aggregated by salt (the color displaying from red to blue). Furthermore, the changes in the dispersity of AuNPs were investigated. As shown in Figure 2, compared with AuNPs, the large aggregate was observed for the AuNPs in the absence of *trans*-zeatin, which resulted in the color change of AuNPs from wine red to blue. By adjusting the relative amount of *trans*-zeatin, we can control the color of AuNPs solution. Therefore, a colorimetric detection method for *trans*-zeatin was established.

**Figure 3:**
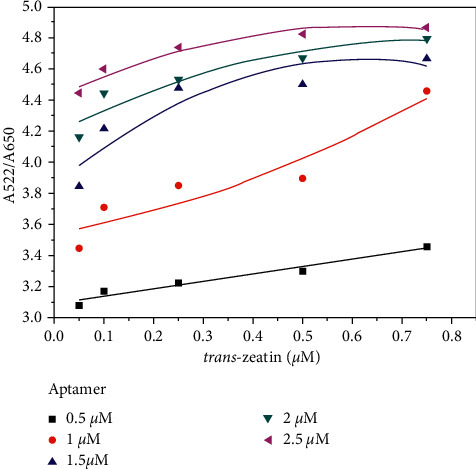
The effects of aptamer concentration on *trans*-zeatin detection. The concentrations of aptamer were 0.5, 1, 1.5, 2, and 2.5 *μ*M, respectively.

In this strategy, the aptamer interacted with *trans*-zeatin or complementary DNA to assemble a rigid duplex due to its positive charge. That is to say, there is a competition between *trans*-zeatin and complementary DNA with aptamer. If the aptamer prior binds with *trans*-zeatin, the rest complementary DNA will spontaneously bind to unmodified AuNPs and stabilize AuNPs effectively. So, it is very important to control the relative concentration of aptamer and *trans*-zeatin.  Figure 3 shows the effects of aptamer concentration on *trans*-zeatin detection. When various concentrations of aptamer between 0.5 *μ*M and 2.5 *μ*M were added into the mixed solution, the color changed, and the ratio of absorbance intensities (A522/A650) were recorded. When the aptamer is 0.5 *μ*M, there is a good linear relationship between the ratios of absorbance intensities with various *trans*-zeatin concentrations. But the trend is more significant when the aptamer is 1 *μ*M, and it is easier to observe the color change of AuNPs. Therefore, 1 *μ*M aptamer was selected for the following research.

Meanwhile, the optimization of the ratio between aptamer and complementary DNA was performed. The studies revealed that the DNA could fold into an unexpected structure due to temperature, pH, pressure, light, and so on. And the aptamer DNA without correct 3D structure could not combine with *trans*-zeatin. Besides, the free end of unexpected structure effected the aggregation of AuNPs. But the complementary DNA could reduce the affection significantly due to the complementary DNA hybridizing with aptamer DNA and the hybridization between complementary DNA and the end of uncorrected folded aptamer. Furthermore, an improvement of salt tolerance of AuNPs was caused by the protection of complementary ssDNA under the high concentration of *trans*-zeatin which was combined with aptamer DNA. So, the complementary DNA can increase significantly the reliability and sensitivity of the detection. The results revealed that either higher or lower concentrations of complementary DNA than aptamer will induce aggregation of AuNPs. Therefore, the concentration of complementary DNA was as same as the aptamer (1 *μ*M) in the study.

**Figure 4:**
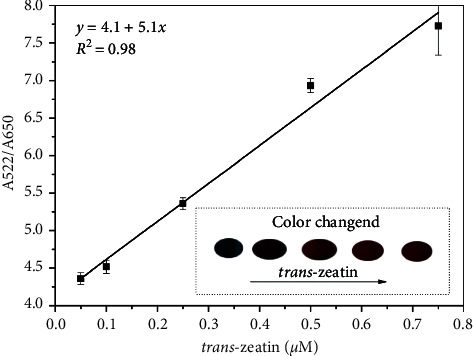
Absorption ratios (A522/A650) as a function of *trans*-zeatin concentration (inset: photographic images with the corresponding solutions).

To detect *trans*-zeatin, absorption ratio of the mixed solution was measured with different concentrations of *trans*-zeatin under optimized conditions.  Figure 4 indicated that the ratio of absorbance intensities increased with the addition of *trans*-zeatin. There is a very good linear relationship between absorption ratio (A522/A650) and the *trans*-zeatin concentration. The correlation coefficient (*R*^2^) is 0.98, and the detection range is from 0.05 to 0.75 *μ*M. The developed sensor has a limit of detection (LOD) which is 0.037 *μ*M, which was calculated according to the 3*δ* criteria. Compared with reported methods for *trans*-zeatin detection by Di et al. (the reported LOD was 0.1 *μ*M) [26], this method has more sensitivity for quantitative analysis of *trans*-zeatin. Furthermore, the color of aptamer-AuNP aggregation could be easily observed by the naked eyes when the concentration of *trans*-zeatin decreased. The color changed from blue-purple to wine-red (see the inset photographic images in Figure 3).

**Figure 5:**
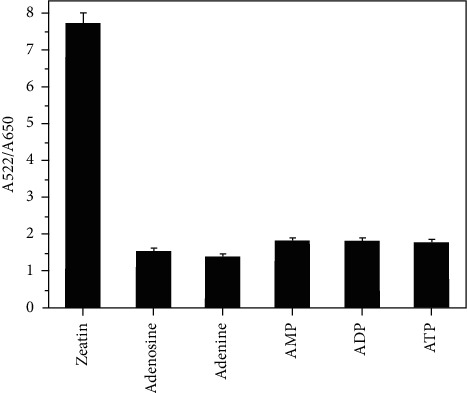
The relative response of the sensing system to different molecules.

The specificity of this method was determined by challenging it with five similar molecules, including 0.75 *μ*M adenosine, adenine, adenosine monophosphate (AMP), adenosine diphosphate (ADP), and adenosine triphosphate (ATP). As shown in Figure 5, the ratio absorption (A522/A650) for the different mixed solutions other than *trans*-zeatin had a little effect. This was due to the weak binding affinity of the aptamer-AuNPs to these molecules and little tendency to form a G-quadruplex. This implied that the proposed colorimetric method is specific for the detection of *trans*-zeatin.

The developed *trans*-zeatin colorimetric sensor based on aptamer-AuNPs was preliminarily applied to the detection of *trans*-zeatin in the leaves of maize. The analysis results are compared with the standard method by HPLC, which are summarized in Table 1. The results show that the recoveries of added *trans*-zeatin in the samples ranged from 98.6% to 112.0% by this method, and the results agree well with standard methods by HPLC. These results demonstrate the potential applicability of this method for the quantitative detection of *trans*-zeatin in the real samples.

Table 2 shows a comparison of this work with those of other zeatin determination reported in the literature. The sensitivity and linear range of this work were not better than those reported in the literature, but its selectivity and accuracy were high. The newly developed colorimetric method is suitable for routine measurement of *trans*-zeatin.

## 4. Conclusions

This work developed a very simple method for the colorimetric detection of *trans*-zeatin in plants. The sensing mechanism is based on the increased stability of AuNPs while binding with released complementary DNA under high concentration of salts. The optical properties of AuNPs are highly distance-dependent, which leads to the direct color change observed by naked eyes. The analytical results showed that the detection limit is 0.037 *μ*M and exhibit good linear relationship from 0.05 to 0.75 *μ*M. Meanwhile, this method exhibits excellent specificity and applicability. The developed method did not utilize any sample pretreatment, organic solvents, enzymatic reactions, or sophisticated instruments, thus overcoming some limitations of more conventional methods.

## Figures and Tables

**Table 1:** Analytical results for *trans*-zeatin in real samples.

Sample	Found origin *trans*-zeatin (*μ*M)	Added *trans*-zeatin (*μ*M)	Found total *trans*-zeatin (*μ*M)	Recoveries (%)	RSD (%, *n* = 3)
1	3.221^a^	0.10	3.327^a^	106.0^a^	0.79^a^
	3.219^b^		3.230^b^	110.0^b^	0.54^b^

2	3.240^a^	0.20	3.464^a^	112.0^a^	0.65^a^
	3.236^b^		3.433^b^	98.5^b^	0.51^b^

3	3.302^a^	0.30	3.608^a^	102.0^a^	0.601^a^
	3.299^b^		3.601^b^	100.1^b^	0.55^b^

4	3.316^a^	0.40	3.711^a^	98.6^a^	0.72^a^
	3.312^b^		3.709^b^	99.3^b^	0.52^b^

^a^Sample was measured by this method. ^b^Sample was measured by HPLC.

**Table 2:** Comparison of some analytical methods used for the determination of zeatin.

Method	Detection limit	Linear range	Reference
Electrochemical aptasensor	16.6 pM	50 pM–50 nM	[30]
Photoelectrochemical apta-biosensor	0.031 nM	0.1 nM–100 nM	[31]
Graphene oxide-protected aptamers	60 nM	0.67–6.0 *μ*M	[32]
A hairpin aptamer and fluorescence method	135 nM	—	[33]
Aptamer-based colorimetric method	0.037 *μM*	0.05–0.75 *μ*M	This work

## Data Availability

The testing data used to support the findings of this study are available from the corresponding author upon request.
